# Beyond Intention: Barriers to Undergoing a Blood Pressure Check in the South-West Shewa Zone, Ethiopia

**DOI:** 10.3390/healthcare12232417

**Published:** 2024-12-02

**Authors:** Bezawit Ketema, Mirgissa Kaba, Mosisa Bekele, Eva Johanna Kantelhardt, Eric Sven Kroeber, Adamu Addissie

**Affiliations:** 1 School of Public Health, College of Health Sciences, Addis Ababa University, Addis Ababa 9086, Ethiopia; mirgissa.kaba@aau.edu.et (M.K.); adamuaddissie@gmail.com (A.A.); 2 Institute for Medical Epidemiology, Biostatistics and Informatics, Martin Luther University, 06097 Halle, Germany; eva.kantelhardt@uk-halle.de (E.J.K.); eric.kroeber@uk-halle.de (E.S.K.); 3 Global Health Working Group, Martin Luther University, 06097 Halle, Germany; mosisabe31@gmail.com

**Keywords:** hypertension, blood pressure, screening, rural health, Ethiopia, public health, intention

## Abstract

Background/Objectives: Hypertension is often asymptomatic, progresses slowly, and leads to multiple secondary diseases. Thus, a regular blood pressure check is recommended. The objective of this study is to assess the intention to undergo a blood pressure check and its associated factors among adults in Southwest Shewa Zone, Ethiopia. Methods: A healthcare-facility-based cross-sectional design was utilized. A standardized questionnaire, adapted from previous research, was administered by trained interviewers. Binary logistic regression analysis was used to determine the factors the intention to undergo blood pressure checks is associated with, utilizing SPSS version 27. Results: Four hundred twenty-one participants provided a complete response, yielding a 99.7% response rate. Of these participants, 153 (36%) had had their blood pressure checked at some point. The vast majority of the study participants (387, 91.9%) did not know the normal blood pressure range. The median score for intention to undergo blood pressure check was 11 (interquartile range 10–13). Female participants were 59% less likely (adjusted odds ratio [AOR] 0.408, 95% confidence interval 0.208–0.801) to intend to undergo a blood pressure check than male participants. Participants in the poorest wealth quintile were 82% less likely (AOR 0.183, 95% CI = 0.063–0.533) to intend to undergo a blood pressure check than those in the richest quintile. Participants who intended to undergo a blood pressure check had a significantly favorable attitude (t = 10.801, *p* < 0.001) and lower perceived behavioral control (t = −2.865, *p* < 0.001) compared with those who had no intention of checking. Conclusion: A high intent to undergo a blood pressure check should prompt healthcare facilities to offer regular blood pressure check-up services. Behavioral change communication interventions should address the attitude and perceived behavioral controls of individuals associated with the intention to undergo a blood pressure check. In doing so, special attention should be given to female and economically disadvantaged populations.

## 1. Introduction

Hypertension, or high blood pressure, is a grave medical condition that can cause brain, heart, kidney, and additional health problems. Globally, an estimated 1.28 billion adults have hypertension, and around two-thirds of these individuals live in low- and middle-income countries [1]. In Africa, the estimated number of people with hypertension is projected to rise to 216.8 million by 2030 (a 66% rise from 2010) [2]. A systematic review and meta-analysis of 38 studies showed a 21.81% hypertension prevalence in Ethiopia [3].

Hypertension may be asymptomatic and progresses very slowly, so it is called a “silent killer” [4,5]. Thus, the World Health Organization (WHO) recommends undergoing a regular blood pressure check. Early detection of high blood pressure through screening can lead to timely treatment, decreasing associated costs and reducing the risk of serious health complications like heart attacks, strokes, and premature death [1]. The United States Preventive Services Task Force (USPST) recommends annual hypertension screening for adults >40 years old and a screening timeframe of 3–5 years for adults aged between 18 and 39 years [6]. In line with international recommendations, the National Guidelines of Ethiopia regarding clinical and programmatic management of major non-communicable diseases also recommend regular hypertension screening for adults ≥ 30 years [7].

Despite the recommendations for hypertension screening, throughout the world, an estimated 46% of adults with hypertension are oblivious to their status [1]. The burden of undiagnosed hypertension is 28.8% in Southern Ethiopia [8] and 21.2% in Southwest Ethiopia [9]. Therefore, the aim of this research is to assess the intention to undergo a blood pressure check and its associated factors among adult primary healthcare facility attendants in the Southwest Shewa zone, Ethiopia, using the theory of planned behavior (TPB). This approach has been applied to a wide variety of health behaviors, including the intention to undergo screening for cervical cancer [10,11,12,13] and clinical breast examination [14]. According to Ajzen’s TPB, intention is influenced by favorable or unfavorable attitudes, subjective norms, and perceived behavioral control, which are shaped by behavioral, normative, and control beliefs, respectively [15]. Even though a target population’s intention to undergo a recommended behavior is a key factor for policy planning and program design, to our knowledge, there have been no studies focusing on the intention to undergo a blood pressure check in this area. Thus, our findings will contribute to filling a knowledge gap among researchers, policymakers, and program designers.

## 2. Materials and Methods

### 2.1. Study Design, Area, and Period

A health-facility-based cross-sectional study was conducted in the Southwest Shewa zone, Oromia, Ethiopia. Southwest Shewa is found 114 km from the capital city, Addis Ababa. Ethiopia’s healthcare system is a three-tier structure. At the primary level, services are delivered through a network of health posts (village-level facilities), health centers, and primary hospitals. Health centers oversee health posts, deliver mainly preventive healthcare services, and do referrals to primary hospitals. Southwest Shewa has 54 health centers, and this study collected data from 12 of them from 10 January 2021 to 10 February 2021.

### 2.2. Study Population

All adults ≥ 30 years, old who were visitors to the selected healthcare facilities during the data collection time were eligible for this study. The rationale for the age cutoff of ≥30 years is based on the Ethiopian National Guidelines on Clinical and Programmatic Management of Major NCDs [7]. Pregnant women and patients diagnosed with hypertension were excluded.

### 2.3. Sample Size Determination

The sample size was calculated using the single population proportion formula with Epi-Info software (version 7). Based on the assumptions of a 95% confidence interval (CI) and a 5% margin of sampling error tolerance (hence there being no similar previous study), we assumed a 50% probability of an intention to undergo a blood pressure check and a 10% non-response rate, and the sample size was calculated at 422.

### 2.4. Sampling Procedures

A total of 12 facilities were chosen randomly from 54 PHFs in Southwest Shewa. The sample was equally distributed among each healthcare facility based on the patient flow in each facility over the previous 10 days, which was similar for each facility and was assumed to be similar throughout the period of data collection. The actual study participants were chosen from the 12 healthcare facilities using systematic random sampling. The sample size interval was computed, and every other nine adults visiting each healthcare facility were approached and interviewed when they exited the facility.

### 2.5. Questionnaire Design and Data Collection

The TPB manual by Ajzen [15] and previous research [16,17] were used to develop this study questionnaire. An elicitation study was conducted prior to developing this study tool in order to identify items for measuring indirect TPB constructs. Sixteen in-depth interviews with participants similar to the target population have generated 11 themes (Table S1).

The outcome variable, the intention to undergo a blood pressure check, was measured using the following items: “How likely is it that you will need blood pressure check service in the next 3 months?”; “In the coming 3 months, how likely is it that you will be checked for blood pressure?”; and “In the next 3 months, how likely is it that you will look for and request blood pressure check service?”. A median split between intending and not intending to undergo a blood pressure check was considered to dichotomize the intention variable [15].

Each TPB construct (attitude, subjective norm, and perceived behavioral control) was measured on a 5-point Likert scale: 1, strongly disagree; 2, disagree; 3, neutral; 4, agree; and 5, strongly agree. Items were clear and unambiguous, each statement addressed only one issue, and appropriate language was used. The items used to measure the direct attitude towards blood pressure check included “a blood pressure check is good”, “a blood pressure check is useful”, and “a blood pressure check feels pleasant.” The items used to measure the direct subjective norm towards blood pressure check were “most people who are important to me think that I should have my blood pressure checked”, “I feel social pressure to have my blood pressure checked”, and “people who are important to me want me to have my blood pressure checked.” The items used to measure direct perceived behavioral control towards a blood pressure check were “a blood pressure check service is easily available”, “a blood pressure check service is easily accessible”, “a blood pressure check is likely expensive”, “I don’t think I am at risk of acquiring high blood pressure”, and “I am aware about hypertension screening service”. We handled TPB dimensions (attitude, subjective norm, and perceived behavioral control) as continuous variables (Table S1).

Considering previous studies [16,17,18,19], knowledge of blood pressure was measured by using nine items. The knowledge-assessing items focused on risk factors, symptoms, and prevention options. The median score was computed, and knowledge was categorized as good or poor (above and below the median score, respectively).

In order to maintain consistency in meaning and content, the structured questionnaire was developed in English, translated by language experts into Afan Oromo, the local language, and then back translated into English. Pilot testing was done on the initial questionnaire draft among a sample of 50 individuals similar to the study population, as per the inclusion criteria of the study. Pilot participants were recruited using the same sampling technique used in this study: systematic random sampling. All demographic categories were represented. Pilot data were analyzed to determine the statistical rigor, identify ambiguities and confusion, assess question order and flow, evaluate response options, estimate time to complete, identify technical issues, and refine the questionnaire. The final questionnaire comprised 10 sections: sociodemographic variables, knowledge about hypertension, past practice of blood pressure checking, intention to undergo a blood pressure check, and direct and indirect TPB constructs. The data were collected by trained data collectors using a structured questionnaire and the Open Data Collection Kit (ODK).

### 2.6. Data Cleaning and Processing

The data gathered using the ODK were verified and exported to SPSS Statistics version 27 for analysis. Items that were negatively specified were inverted prior to analysis. An analysis was conducted to assess the internal consistency of the items measuring the direct theory of planned behavior (TPB) constructs. Cronbach’s alpha was >0.7 for each TPB dimension—0.708 for direct attitude, 0.756 for direct subjective norm, and 0.762 for direct perceived behavioral control—confirming the internal consistency of the questionnaire. Simple correlations among the direct and indirect measures of the similar TPB constructs were calculated to verify the validity. Prior to performing any type of analysis, the normality, homogeneity of variance, and multicollinearity of the data were examined. The variance inflation factor was <10, indicating a lack of multicollinearity.

### 2.7. Data Analysis

Data were summarized utilizing frequency, proportion, central tendency, and dispersion. Independent-sample t-tests were employed to compare the TPB constructs of individuals who intend to and do not intend to undergo a blood pressure check. The Holm-Bonferroni method was used to adjust for significance levels to 0.0167, 0.025, and 0.05 for multiple comparisons and to reduce the risk of type I error.

To identify factors associated with the intention to undergo a blood pressure check, a binary logistic regression model was employed. A *p*-value of less than 0.25 in the bivariate analysis [20], multicollinearity, clinical significance, and maximum number of variables a model can tolerate were considered to enter variables in the multiple logistic regression model. Statistical significance for the multivariable logistic regression analysis was set at *p* < 0.05. The Hosmer–Lemeshow goodness-of-fit test was run to confirm that the model adequately fit the data.

## 3. Results

### 3.1. Sociodemographic Characteristics of Study Participants

In this study, 421 participants provided a complete response, yielding a 99.7% response rate. Study participants’ ages ranged from 30 to 81 years, with a mean of 44.7 years and a standard deviation of 10.2 years. More than half (n = 247, 58.7%) of the study participants were women. Most of the participants (n = 364, 86.5%) were married. Almost half (n = 184, 43.7%) were Orthodox Christians. In terms of education and occupation, 153 (36.3%) participants could not read or write, and 221 (52.5%) participants were farmers. The majority of spouses (n = 266, 63.2%) were farmers and could mostly read and write without formal education (n = 123, 29.5%; Table 1).

### 3.2. Knowledge About Blood Pressure and Past Blood Pressure Check Practice

**Figure 1:**
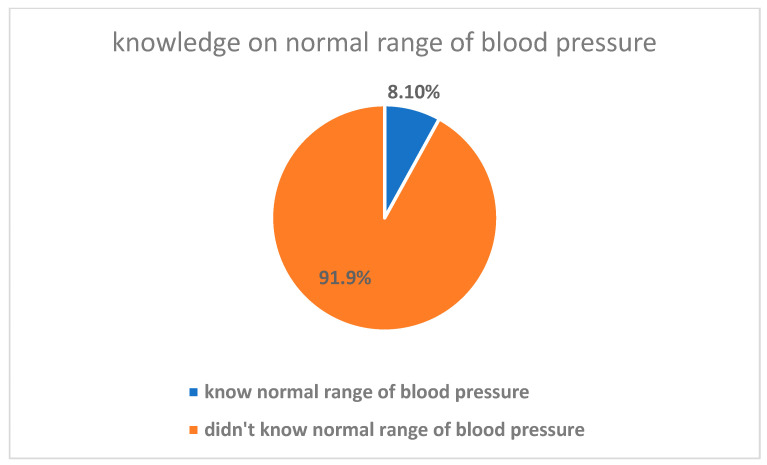
Knowledge of normal range of blood pressure among study participants.

Most of the participants (n = 278, 66%) stated that high blood pressure cannot be transmitted. Moreover, most of the participants (279, 66%) knew at least one risk factor: 156 (37%) mentioned high fat and 102 (24%) mentioned heredity. The majority of the participants (n = 300, 71%) correctly mentioned at least one sign and symptom; 266 (63%) participants mentioned headache as a sign and symptom of high blood pressure, while 121 (28.7%) participants said they were unsure. Very few of the study participants (34, 8.1%) knew the normal range of blood pressure, while most of them (376, 89%) said they did not know it (Figure 1). When asked about the consequences of untreated high blood pressure, 383 (91%) participants were aware of at least one complication. Death was mentioned as a common complication by 258 (61%) participants.

Regarding prevention of high blood pressure, 336 (80%) participants correctly mentioned at least one prevention method. Half of the study participants mentioned reduced fatty food intake as one of the high blood pressure prevention measures. Furthermore, minimizing salt intake and taking antihypertensive were mentioned by 185 (44%) and 128 (30%) participants, respectively. Regarding the aim of blood pressure screening for healthy individuals, slightly more than half of the participants mentioned detecting hypertension early, while more than a quarter of the participants did not know the aim.

Overall, the majority of the participants (n = 321, 76%) had good knowledge about blood pressure. Moreover, 153 (36%) participants had their blood pressure checked at some point. The two most common justifications were that it was the usual practice at the clinic (93%) and that it was a health professional’s recommendation (58%; Table 2).

### 3.3. Intention to Undergo Blood Pressure Check

The median intention to undergo a blood pressure check score was 11 (interquartile range [IQR] 10–13). Among the study participants, 224 (53%, 95% CI 48%–58%) intended to undergo a blood pressure check in the next 3 months. The participants who intended to undergo a blood pressure check had significantly favorable attitudes (t = 10.801, *p* < 0.001) and lower perceived behavioral control (t = −2.865, *p* < 0.001). However, there was no difference in subjective norms (Table 3).

### 3.4. Associated Factors with Intention to Undergo Blood Pressure Check

Female participants were 59% less likely (adjusted odds ratio [AOR] 0.408, 95% CI = 0.208–0.801) to intend to undergo a blood pressure check than male participants. Housewives were 3.4 times more likely (AOR 3.400, 95% CI 1.653–6.992) to intend to undergo a blood pressure check than farmers. The participants in the poorest wealth quintile were 82% less likely (AOR 0.183, 95% CI 0.063–0.533) to intend to undergo a blood pressure check than those in the richest quintile. A favorable attitude and lower perceived behavioral control would increase the intention to undergo a blood pressure check. Each unit increase in the attitude score would increase the odds of intending to undergo a blood pressure check by 3.7-fold (AOR 3.711, 95% CI 2.315–5.947). The odds of intending to undergo a blood pressure check would decrease by 18% (AOR 0.822, 95% CI 0.690–0.981) per unit increase in perceived behavioral control (Table 4).

## 4. Discussion

In this study, 53% of the participants intended to undergo a blood pressure check in the next 3 months. Though there are no prior studies that have focused on the intention to undergo a blood pressure check/hypertension screening in Ethiopia, this finding is consistent with studies done on the intention to undergo screening for other non-communicable diseases in the same country [12,13,14,21].

Female participants in this study were less likely to intend to undergo a blood pressure check than male participants. This disparity might be explained by the knowledge gap between the two sexes, as 86% of men, compared to 69% of women in this study, demonstrated good blood pressure knowledge. The lower intention to undergo blood pressure checks among females than males may lead to the high prevalence of undiagnosed hypertension among women. Thus, interventions targeting women to increase their intention to undergo a blood pressure check are required. This study found that participants in the poorest wealth quintile were 82% less likely (AOR 0.183, 95% CI = 0.063–0.533) to intend to undergo a blood pressure check than those in the richest quintile. This difference could be attributed to the knowledge disparity, as 94% of the wealthiest group and 75% of the poorest group exhibited good blood pressure knowledge. The reason behind more housewives intending to undergo a blood pressure check could also be the difference in blood pressure knowledge among the two demographic groups; a higher percentage of housewives (79%) possessed good blood pressure knowledge compared to farmers (71%).

In this study, we found that the odds of intending to undergo a blood pressure check increased by 3.7 fold per unit in the attitude score. This significant relationship between attitude and intention has been repeatedly indicated in different health behavior studies, mainly those that have focused on non-communicable disease screening behaviors, including the intention to undergo clinical breast examination [22] and cervical cancer screening [10].

Relatively few of this study’s participants (13%) knew that hypertension could be asymptomatic, and only 36% of the participants had had their blood pressure checked at some point in their life. This percentage is lower than that found in studies in Lebanon [23], Kenya [24], and Nigeria [19], where 45%, 65%, and 42% of the study participants, respectively, had had their blood pressure checked at some point. The low blood pressure check-up practice in this study setting indicates that a higher prevalence of undiagnosed hypertension among the study population is more likely. Consistently, the authors of a systematic review on the epidemiology of hypertension in Ethiopia found that 37%–78% of patients with hypertension in Ethiopia were not aware of their blood pressure condition [25]. Thus, behavior change communication interventions are highly required to increase the knowledge and practice of blood pressure checks in the study setting, thereby lowering the high proportion of undiagnosed hypertension in the country at large.

The two most common justifications for having hypertension screening were that it was the usual practice at the clinic (93%) and a recommendation by a health professional (58%). Likewise, a study conducted in the Arsi zone, Oromia, Ethiopia, indicated that receiving a health professional’s recommendation was significantly associated with self-monitoring of blood pressure [26]. In Ethiopia, a health professional’s recommendation is also an influencing factor for performing a breast self-examination [27]. Hence, recommendations by health professionals represent a good approach to increasing any screening practice. Thus, we encouraged involving healthcare professionals to give behavioral recommendations like NCD screening in behavioral change communication interventions for promoting NCD screening.

Almost all of the study participants (91.9%) did not know the normal blood pressure range. According to a study done in Gondar Comprehensive Specialized Hospital in Ethiopia, among patients with hypertension, who are likely to know better than the undiagnosed population, 38% of the respondents did not know the normal blood pressure level [8]. In terms of risk factors, only 37% and 24% of the study participants mentioned high fat intake and heredity/genetic background, respectively, as risk factors for hypertension, and 34% did not know any risk factors. However, in the Gondar study, most of the respondents were aware of the negative impact of cigarette smoking and alcohol drinking (79% and 88%) respectively; [8].

This study’s findings, in general, showed a gap in knowledge and practice regarding blood pressure and blood pressure checks, which indicates a need for educational intervention. Moreover, the findings of this study can be used to inform the design of interventions that promote the intention to undergo blood pressure checks. The policy implications of this study can also be relevant for other low-income countries that have similar contextual factors to this study setting.

### Strengths and Limitations

The strengths of this study include the use of validated measures and the exploration of salient beliefs through elicitation. Looking at the participants’ backgrounds, all groups represented eco-epidemiological settings. Therefore, we assumed the findings could be generalized beyond the specific context of this study, considering the limitations of a facility-based cross-sectional design. Additionally, we were unable to determine whether the reported intention to undergo a blood pressure check translated into actual procedures.

## 5. Conclusions

Although there have been very limited studies focusing on the intention to undergo a blood pressure check, we found a lower intention to undergo a blood pressure check compared with other NCD screening services in this study setting. A higher wealth index, the male sex, a positive attitude, and low perceived behavioral control had a positive association with the intention to undergo a blood pressure check. We recommend expanding blood pressure check-up services together with social and behavioral change communication (SBCC) interventions to ensure health facilities have trained nurses who can check blood pressure. In doing so, special attention should be given to female and economically disadvantaged populations. Thus, the SBCC intervention should ensure its accessibility, understandability, and usability by these populations.

## Figures and Tables

**Table 1:** Sociodemographic characteristics of the study participants.

Characteristics	Response Category	Frequency (N = 421)	Percent (%)
Sex	Female	247	58.7
	Male	174	41.3
Religion	Orthodox	184	43.7
	Muslim	108	25.7
	Protestant	129	30.6
Marital status	Married	364	86.5
	Widowed	41	9.7
	Never married	16	3.8
Educational status	Cannot read and write	153	36.3
	Read and write without formal education	105	24.9
	Primary education	117	27.8
	Secondary education and above	46	10.9
Occupation	Government/non-governmental organization employee	17	4.0
	Housewife	127	30.2
	Merchant	56	13.3
	Farmer	221	52.5
Spouse occupation	Government/non-governmental organization employee	36	8.6
	Merchant/housewife	62	14.7
	Farmer	266	63.2
Spouse educational status	Cannot read and write	106	25.2
	Read and write without formal education	123	29.2
	Primary education	84	20.0
	Secondary education and above	51	12.1

**Table 2:** Knowledge about blood pressure and past blood pressure check practices (n = 421).

Characteristics	Response Category	Frequency (N = 421)	Percent (%)
High blood pressure can be transmitted	Yes	52	12.4
	No	278	66
	Don’t know	91	21.6
Risk factors for high blood pressure (multiple responses are possible)	Hereditary/genetic background	102	24.23
	Smoking	84	19.95
	Obesity	7	1.7
	High fat intake	156	37
	Excess alcohol intake	72	17.1
	Don’t know	142	33.7
	Other	19	4.5
Signs and symptoms of high blood pressure (multiple responses are possible)	Asymptomatic	53	12.6
	Headache	266	63.2
	Palpitations	123	29.2
	Poor vision	66	15.7
	Dizziness	57	13.5
	Don’t know	121	28.7
	Other	7	1.68
Normal blood pressure range	Less than 120/80 mmHg	34	8.1
	Less than 130/90 mmHg	9	2.14
	Less than 160/100 mmHg	2	0.48
	Don’t know	376	89.31
Complications if blood pressure remains untreated (multiple responses are possible)	Stroke	139	33
	Heart failure	131	31.1
	Kidney failure	69	16.4
	Loss of sight	34	8.1
	Death	258	61.3
	Don’t know	38	9
Prevention/control measures for high blood pressure (multiple responses are possible)	Minimize salt intake	185	43.9
	Reduce fatty foods	211	50.1
	Avoid excess alcohol	123	29.2
	Avoid smoking	84	20
	Regular exercise	49	11.6
	Taking antihypertensive	128	30.4
	Measuring blood pressure	39	9.3
	Don’t know	85	20.2
	Other	16	3.84
Aims of a blood pressure check for a healthy individual (multiple responses are possible)	To detect hypertension at an early stage	224	53.2
	To prevent complication	182	43.2
	Don’t know	115	27.3
	Other	4	0.96
Have had a blood pressure check	No	268	66.7
	Yes	153	36.3
Reasons to undergo blood pressure check (multiple responses are possible) (n = 153)	Health professional’s recommendation	88	57.5
	It is a standard care at the clinic	93	61
	Other	4	2.6

**Table 3:** The theory of planned behavior (TPB) constructs among the participants intending or not intending to undergo a blood pressure check.

TPB Construct	Intend to Undergo a Blood Pressure Check		Do Not Intend to Undergo a Blood Pressure Check		*t*	*p*	95% Confidence Interval
	Mean	SD	Mean	SD			
Attitude	4.372	0.671	3.819	0.541	10.801	<0.001	−0.314 0.050
Perceived behavioral control	8.268	1.715	8.934	1.329	−2.865	<0.001	−0.963 −0.369
Subjective norm	2.789	1.129	2.921	0.684	−2.529	0.155	0.435 0.671

**Table 4:** Factors associated with the intention to undergo a blood pressure check.

Characteristic	Response Category	Intention		Crude Odds Ratio (95% Confidence Interval)	*p*	Adjusted Odds Ratio (95% Confidence Interval)	*p*
		No (n)	Yes (n)				
Sex	Female	131	116	0.541 (0.364–0.803)	0.002	0.408 (0.208–0.801)	0.009
	Male	66	108	1		1	
Educational status	Cannot read and write	79	74	0.369 (0.180–0.755)	0.006	0.848 (0.254–2.828)	0.789
	Read and write only	55	50	0.358 (0.170–0.756)	0.007	1.072 (0.336–3.417)	0.907
	Primary education	50	67	0.528 (0.252–1.105)	0.090	1.235 (0.410–3.716)	0.707
	Secondary and above	13	33	1		1	
Occupation	Government/non-governmental organization employee	6	11	1.600 (0.572–4.479)	0.371	1.620 (0.314–8.375)	0.565
	Housewife	55	72	1.143 (0.736–1.773)	0.552	3.400 (1.653–6.992)	0.001
	Merchant	33	23	0.608 (0.336–1.102)	0.101	1.000 (0.395–2.536	0.999
	Farmer	103	118	1		1	
Spouse’s occupation	Government/non-governmental organization employee	12	24	1.827 (0.878–3.805)	0.107	0.396 (0.139–1.133)	0.084
	Merchant/housewife	34	28	0.752 (0.432–1.311)	0.315	0.539 (0.234–1.241)	0.146
	Farmer	127	139	1		1	
Spouse’s educational status	Cannot read and write	43	63	0.501 (0.239–1.050)	0.067	1.353 (0.452–4.048)	0.589
	Read and write only	71	52	0.251 (0.121–0.517)	<0.001	0.657 (0.236–1.830)	0.422
	Primary education	46	38	0.283 (0.132–0.606)	0.001	0.529 (0.196–1.432)	0.210
	Secondary and above	13	38	1		1	
Wealth quintile	Poorest	45	39	0.395 (0.210–0.744)	0.004	0.183 (0.063–0.533)	0.002
	Q2	50	33	0.301 (0.159–0.570)	0.000	0.150 (0.053–0.425)	<0.001
	Q3	43	41	0.435 (0.231–0.817)	0.010	0.169 (0.058–0.487)	0.001
	Q4	33	50	0.691 (0.365–1.309)	0.257	0.271 (0.101–0.729)	0.010
	Richest	26	57	1		1	
Ever had blood pressure checked	No	142	126	0.498 (0.331–0.749)	0.001	0.787 (0.454–1.363)	0.392
	Yes	55	98	1		1	
Attitude per unit increase ^a^				4.160 (2.914–5.940)	<0.001	3.711 (2.315–5.947)	<0.001
Subjective norm per unit increase ^a^				0.863 (0.703–1.058)	0.155	1.141 (0.826–1.577)	0.424
Perceived behavioral control per unit increase ^a^				0.757 (0.665–0.861)	<0.001	0.822 (0.690–0.981)	0.029

^a^ Continuous variable.

## Data Availability

The data presented in this study are available on request from the corresponding author.

## Supplementary Materials

The following supporting information can be downloaded at https://www.mdpi.com/article/10.3390/healthcare12232417/s1. Table S1: The theory of planned behavior constructs and the intention to undergo a blood pressure check.
