# Supplementary material for: Beyond Intention: Barriers to Undergoing a Blood Pressure Check in the South-West Shewa Zone, Ethiopia
Source: Healthcare (Basel). 2024 Dec 2;12(23):2417. doi: 10.3390/healthcare12232417 (PMC11641526; doi:10.3390/healthcare12232417)
Supplement: Supplementary file 1 [file healthcare-12-02417-s001.zip › healthcare-3314809-supplementary.pdf]

Table S1. Theory of Planned Behavior constructs on intention to undergo blood pressure check

Section I Theory of Planned Behavior constructs towards blood pressure check							
A. Attitude towards blood pressure check							
1. Direct attitude towards blood pressure check							
Sr. no.	Questions	Response categories					
401	blood pressure check is ___	Bad	1	2	3	4	5	Good
402	Undertaking blood pressure check is ________	Worthless	1	2	3	4	5	Useful
2. Indirect attitude towards blood pressure check								
Behavioral beliefs towards blood pressure check								
404	Blood pressure check______ helps to know the health status	Unlikely	1	2	3	4	5	Likely
405	blood pressure check ________prevent development of blood pressure complication	Unlikely	1	2	3	4	5	Likely
406	blood pressure check______induces stress	Unlikely	1	2	3	4	5	Likely
Outcome evaluation of beliefs towards blood pressure check								
408	Knowing the health status is _______	Extremely undesirable	-2	-1	0	1	2	Extremely desirable
409	Preventing blood pressure complication is ________	Extremely undesirable	-2	-1	0	1	2	Extremely desirable
410	Stress due to blood pressure check is _____________	Extremely undesirable	-2	-1	0	1	2	Extremely desirable
B. Subjective norm towards blood pressure check								
1. Direct subjective norm towards blood pressure checking								
412	Most people who are important to me think that I ______ Be checked for blood pressure	Should not	1	2	3	4	5	Should
413	I feel under social pressure to blood pressure checking	Strongly disagree	1	2	3	4	5	Strongly agree
414	People who are important to me want me to be checked for blood pressure.	Strongly disagree	1	2	3	4	5	Strongly agree
2. Indirect subjective norm towards blood pressure check								
Normative beliefs towards blood pressure check								
416	My family thinks I _____be checked for blood pressure	Should not	-2	-1	0	1	2	Should
417	My community leaders ______ of my blood pressure checking	Disprove	-2	-1	0	1	2	Approve
418	Health care providers ______ of my blood pressure checking	Disprove	-2	-1	0	1	2	Approve
Motivation to comply with normative beliefs towards blood pressure check								
419	My family's approval of my practice is important to me	Not at all	1	2	3	4	5	Very much

420	what my community leaders think I should do matters to me	Not at all	1	2	3	4	5	Very much
421	what health care providers think I should do matters to me	Not at all	1	2	3	4	5	Very much
C. Perceived behavioural control towards blood pressure checking								
1. Direct perceived behavioral control towards blood pressure check								
423	For me blood pressure checkis ______	Easy	1	2	3	4	5	Difficult
424	The decision to take blood pressure checkis beyond my control	Strongly disagree	1	2	3	4	5	Strongly agree
425	Whether I take blood pressure checkor not is entirely up to me	Strongly disagree	1	2	3	4	5	Strongly agree
2. Indirect perceived behavioral control towards blood pressure check								
Control beliefs towards blood pressure check								
426	blood pressure checkservice is ______ available	Not easily	1	2	3	4	5	Easily
	blood pressure checkservice is ______ accessible	Not easily	1	2	3	4	5	Easily
427	Blood pressure checkis ______expensive	Unlikely	1	2	3	4	5	Likely
428	I don't think I am at risk of acquiring blood pressure	Strongly disagree	1	2	3	4	5	Strongly agree
429	I _____aware about blood pressure checkservice	Am not	1	2	3	4	5	Am
perceived power to influence the behaviour towards blood pressure check								
431	If blood pressure checkis not easily available, i am ___to be checked for blood pressure	Less likely	-2	-1	0	1	2	More likely
	If blood pressure checkis not easily accessible, i am ___to be checked for blood pressure	Less likely	-2	-1	0	1	2	More likely
432	If blood pressure checkis expensive, i am___ to be checked for blood pressure	Less likely	-2	-1	0	1	2	More likely
433	Even if i think i am not at risk of acquiring blood pressure, i am ____to be checked for blood pressure	Less likely	-2	-1	0	1	1	More likely
434	Even if i am not aware about blood pressure checkservice, i am ___ to be checked for blood pressure	Less likely	-2	-1	0	1	1	More likely

D. Intention to blood pressure checkin the next 3 months						
		Very unlikely	Unlikely	undecided	Likely	Very likely
436	How likely is it that you will need blood pressure checkservices in the next 3 months?	1	2	3	4	5

**437**	**In the coming 3 months, how likely is it that you will be checked for blood pressure?**	**1**	**2**	**3**	**4**	**5**
**438**	**In the next 3 months, how likely is it that you will look for and request blood pressure checkservices?**	**1**	**2**	**3**	**4**	**5**
**439**	**How likely is that you will think to screen for blood pressure for the next 3 month?**	**1**	**2**	**3**	**4**	**5**
